# Impact of Work Motivation on Occupational Health in Healthcare Workers

**DOI:** 10.3390/healthcare11233056

**Published:** 2023-11-28

**Authors:** Antonella D’Alleva, Angela Coco, Gilda Pelusi, Chiara Gatti, Pietro Bussotti, David Lazzari, Massimo Bracci, Andrea Minelli, Beatrice Gasperini, Emilia Prospero

**Affiliations:** 1 Department of Biomedical Sciences and Public Health, Università Politecnica delle Marche, 60126 Ancona, Italym.bracci@staff.univpm.it (M.B.);; 2 School of Nursing Science, Università Politecnica delle Marche, 60121 Ancona, Italy; s1091205@studenti.univpm.it (A.C.); g.pelusi@staff.univpm.it (G.P.); 3 Heart Surgery Department, Azienda Ospedaliero Universitaria, di Ancona, 60126 Ancona, Italy; chiara.gatti@ospedaliriuniti.marche.it; 4 School of Psychology, European University of Rome, 00163 Rome, Italy; pietro.bussotti@unier.it; 5 Italian Society of Psychoneuroendocrineimmunology (SIPNEI), 00195 Rome, Italy; davidlazzari888@gmail.com; 6 National Council of the Order of Psychologists, 00198 Rome, Italy; 7 Department of Biomolecular Sciences, Urbino University, 61029 Urbino, Italy; andrea.minelli@uniurb.it; 8 Azienda Ospedaliera Ospedali Riuniti Marche Nord, 61032 Fano, Italy

**Keywords:** healthcare system, autonomous motivation, controlled motivation, occupational well-being, burnout, work-related stress

## Abstract

Objectives: The present cross-sectional study investigated, in a group of Italian healthcare workers (HCWs), the association between work motivation and occupational health and the impact of socio-demographic and job-related variables on this association. Methods: A total of 656 subjects (nurses, technicians, midwives and physiotherapists) completed the survey. Linear regression models were used to correlate motivation types (by Scale of Motivation At Work) with health indicators (general health, depression, professional exhaustion, satisfaction and turnover intention) and burnout’s subscales (emotional exhaustion, depersonalization and reduced professional achievement). Findings: Autonomous motivation correlated positively with general health and work satisfaction and negatively with depression, exhaustion and turnover intention. Scoring high on intrinsic/integrated regulation was associated with better health and job satisfaction and with turnover intention, depression and emotional exhaustion. Controlled motivation, demotivation and external regulation nourished burnout’s indicators, while autonomous motivation was protective. Operating in intensive care or surgical areas negatively affected general health; working as a nurse manager or midwife increased one’s depressive risk and reduced satisfaction; being older than 60 increased emotional exhaustion and turnover intention; having a master’s degree protected from exhaustion and depression. Implications: Collectively, our findings extend evidence on the role of work motivation in shaping occupational health and underline the importance for healthcare organizations of promoting actions to reinforce autonomous motivation at work.

## 1. Introduction

The efficiency of the healthcare system requires healthy and productive healthcare workers (HCWs): in fact, it is well known that poor occupational health among HCWs poses serious risks to the health trajectories of HCWs themselves, and it reduces the quality of care delivered to the patients [1,2]. Occupational health, as defined by Avallone and Paplomatas [3], is a complex of diverse cultural and organizational processes that help nourish coexistence in work contexts by promoting and maintaining the psycho-physical and social well-being of workers. Such state of health can be characterized by positive indicators of well-being (job satisfaction, belonging, desire to go to work and trust) and by negative ones (desire to change jobs, a sense of irrelevance and the absence of involvement), which imply malaise and disinterest. Excessive emotional and physical demands often associated with healthcare work may hamper the efforts of health professionals to meet the users’ needs, thus resulting in the tendency to cognitively and sentimentally distance themselves from work and sometimes leading to the decision to abandon it [4,5,6,7].

Prior research [8,9] revealed that occupational health among HCWs is associated with their motivation at work. Motivation is a multifaceted, highly dynamic mental construct encompassing a set of bio-psycho-social dimensions (emotions, life experiences and personal relationships) which activate, direct and support human behavior [10]. As an integral part of the relationship between subject and organization [11], motivation at work can be seen as the drive leading the individual to engage diligently in their job, which arises from the manifestation of a need, of a problem, that generates an urge to solve it [10].

Motivation and needs have been traditionally treated as interwoven concepts. Maslow [12] suggested that motivation originates from needs, which are distributed in a pyramidal order (so that no high-level need can ever be motivating if one of a lower order is not satisfied first); in particular, the motivation at work is part of high-order, esteem and self-realization needs. Herzberg’s “Dual Theory” [13] grounded professional motivation on two principal sets of factors: hygienic factors—such as one’s work context, salary, relationships and safety conditions—and directly motivating factors—such as recognition, responsibility and professional growth. McClelland [14] described three fundamental needs, and thus motivations, in human action: motivation for success, for power and for affiliation. According to the “Self-Determination Theory” (SDT) by Deci and Ryan [15,16], people are motivated to grow and change for innate needs; in particular, self-determination is thought as a central concept in the construct of motivation—as a natural propensity to determine oneself independently and freely. In SDT, it is the type of motivation that counts, rather than the quantity, as the most important predictor of outcomes in life. 

Fundamental to SDT is the distinction between different forms of human motivation: the “autonomous” motivation involves a voluntarily undertaken, self-determined behavior, which is perceived as consistent with intrinsic personal goals; the “controlled” motivation, by contrast, involves a behavior forced by external pressure to the self; finally, “demotivation” is the absence of an intention to act. Furthermore, it is necessary to distinguish between motivation and regulation: both concepts indicate forms of psychological energy that direct behavior, but in a superior and subordinate way, respectively. Schematically, macro-categories of motivations (demotivation and autonomous and controlled motivation) encompass distinct sub-categories that reflect different types of regulation (intrinsic, integrated, identified, introjected and external). Autonomous motivation includes intrinsic, integrated and identified regulation types: intrinsic regulation reflects engaging in activities that one finds inherently satisfying through interest and enjoyment; integrated regulation is present when activities are personally meaningful and consistent with a person’s core values; identified regulation is present when the individual identifies himself with the behavior carried out because it is personally important and contributes to the world. Controlled types of motivation include introjected and external forms of regulation: introjected regulation is driven by self-worth contingencies involving the standards or goals of others that have been only partially internalized, and it is associated with avoiding feelings of guilt and shame; external regulation is based on the experience of feeling controlled by external tangible rewards, such as monetary payments and bonuses, or by avoiding punishments. In SDT, motivation/regulation types fall along a self-determination, internalization continuum ranging gradually from feeling externally controlled (least autonomous or most demotivated) at one extreme to feeling autonomous and self-determined with respect and authenticity to goals. 

Evidence shows that individuals with greater autonomous motivation are generally healthier and more productive than those whose motivation is primarily controlled [17]. In fact, autonomous forms of work motivation were found to be associated with occupational health indicators such as work satisfaction, psycho-physical well-being and lower turnover rates [9]. In contrast, controlled forms of work motivation were found to be negatively associated (or unrelated) to occupational health indicators [18,19]. As for HCWs, in particular, a recent survey conducted on a population of more than 3500 physicians has revealed that doctors with high levels of autonomous motivation at work were those who reported a better occupational health status [4]. Among HCWs, work discomfort is thought to increase the risk of developing burnout syndrome [20], which has been defined as a chronic response to stress in the workplace, causing work-related pressure that is out of control and unable to be sidestepped [21], including three main components [22,23]: “emotional exhaustion” (EE), “depersonalization” (DE) and “reduced professional achievement” (RPA). Among nurses, which represent the largest group of healthcare workers, high incidences (from 30% to more than 60%) of burnout and intention to leave have been reported [20,24,25]. Given the link between occupational health and work motivation among HCWs, the implementation of effective work environments, as well as the promotion of good levels of occupational health, necessarily require the identification and targeting of factors that are related to work motivation in healthcare settings. Given the potential impact of work motivation on HCWs’ well-being [1,26]], we acknowledged the importance of investigating the relationships linking different forms of motivation (autonomous, controlled and demotivation) and related regulation types with the occupational health status in HCWs. 

The main purpose of the present study was to evaluate, in a group of HCWs working at the “Ospedali Riuniti” of Ancona (Italy), (i) if different types of motivation (and their related forms of regulation) display differential associations with occupational health indicators and (ii) the possible impact of socio-demographic and occupational factors on these associations. Here, we hypothesized that: (i) autonomous work motivation/regulation would be associated with better occupational health indicators and (ii) different socio-demographic and professional variables would show a differential impact on this association.

## 2. Materials and Methods

### 2.1. Study Design and Sample

#### 2.1.1. Study Design

This is a cross-sectional study, conducted between June 2019 and February 2020, recruiting healthcare professionals operating at “Ospedali Riuniti” of Ancona (Italy). 

#### 2.1.2. Study Sample

The study population was composed of 1849 healthcare workers. Most participants performed activities in clinical departments, particularly medical (hematology, oncology, pneumology, pediatrics and neurology), surgical (surgical rooms and surgical wards) and intensive ones (emergency wards and intensive care units for adults and children). Some respondents operated in the services, embracing different areas (dialysis, day hospitals and the clinical analysis laboratory). All workers were enrolled during the periodic medical examinations required by Italian Law. As part of the standard occupational health surveillance, the study needed no formal approval by the local ethics committee. Nevertheless, the committee was consulted, and it granted an informal authorization. Workers provided their consent after receiving information about the purpose and procedures of the study, which was conducted according to the Helsinki Statement of Ethical Standards. The data were collected with the authorization of the Hospital Direction. Nurse managers were asked to actively collaborate in advertising the study and recruiting participants after attending a preliminary meeting, in which the aims and methodology of the study were thoroughly presented.

#### 2.1.3. Exclusion Criteria

Subjects affected by chronic diseases and subjects reporting severe insomnia or particularly stressful events in the previous 6 months (serious accidents, bereavement, separations and house removals) were excluded from the study.

#### 2.1.4. Instrument and Survey

The anonymous and self-administered survey, validated by calculating the “Cronbach’s α” coefficient, was an Italian web-based format questionnaire, and it was sent to healthcare professionals via business or personal e-mail addresses. At the beginning, the respondents were given a brief explanatory introduction to the main study-related topics and the legislative reference regarding personal data handling and protection (European Regulation 679/2016), to which the participants consented by completing the form. After the first sending, some reminders were sent: where further necessary, a researcher delivered the questionnaire in paper format to the professionals available. The survey included 58 questions, grouped into four sections:

1. Socio-demographic and professional data (gender, age, profession, workplace and years of service);
2. The Scale of Motivation At Work (MAWS) [9,11] explored the main dimensions of autonomous and controlled motivations, as well as their absence (demotivation), in a work setting. It was composed of 18 items, grouped into four subscales: the intrinsic and integrated regulations (items 1–5), the introjected regulations (items 9–12) and the external regulations (items 13–18). Items 6–8 referred specifically to demotivation. The analysis of the identified type of regulation was stackable with the intrinsic one [11]. Answers were given on a Likert scale ranging from 1 (“very untrue for me”) to 7 (“very true of me”). For the first subscale (items 1–5), a score of 7 stands for the highest level of autonomous motivation, while a score of 1 stands for the lowest; in the demotivation subscale (items 6–8), a score of 1 means the absence (lowest levels) of demotivation; for controlled motivation and related introjected and external regulation types (items 9–18), rating a score of 1 means being the lowest in such subscales, thus reflecting a favorable motivation/regulation condition at work.
3. The Maslach Burnout Inventory (MBI) [23] was made of 22 items, exploring the burnout subcategories of “emotional exhaustion” (EE), “depersonalization” (DE) and “reduced professional achievement” (RPA). The answers were given on a six-point Likert scale (0 = never experienced, 6 = always experienced). The level of burnout was classified as low (≤14 for EE, ≤3 for DE and ≥37 for RPA), medium (15–23 for the EE, 4–8 for the DE and 30–36 for the RPA) or high (≥24 for EE, ≥9 for ED and ≤29 for RPA) [27].
4. The multiple-choice questions explored the occupational health (with scores ranging from 1, corresponding to the response option “better than one year ago”, to 5, corresponding to “worse than one year ago”) [28] and its indicators: the risk of depression and emotional exhaustion (with scores ranging from 1, “never felt”, to 4, “always felt”) [29]; work satisfaction (with response options ranging from 1, “completely unsatisfied”, to 5, “completely satisfied”) [30]; and the intent or lack thereof to quit the current work setting or the job in two years (1 = yes; 2 = not) [31].

### 2.2. Data Analysis and Statistics

The data were analyzed using descriptive and inferential statistics. The weight of the different variables was obtained from the construction of two linear regression models. The first one correlated the types of motivation/regulation (autonomous, intrinsic, integrated; controlled, introjected, external; demotivation) with each of the six indicators of occupational health: self-reported general health, risk of depression, professional exhaustion, job satisfaction and intention to leave the unit care and the profession within 2 years. The other one associated the different motivation/regulation types with the three sub-categories of burnout (EE, DE and RPA), explored in the low, medium and high levels. In each linear regression, we distinguish a Model 1 and a Model 2: the first one refers to the association of macro-categories of motivation (autonomous and controlled and demotivation); the second one refers to the subordinate categories of regulation types (intrinsic, integrated, introjected and external). All regression models were adjusted for the socio-demographic and professional characteristics of the sample: role (nurse, physiotherapist, laboratory and radiology technician, perfusionist and midwife), training and educational attainment (regional school, bachelor’s degree and master’s degree, first-level university master, second-level university degree and doctorate), gender (male or female), age (20–29, 30–39, 40–49, 50–59 and >60), setting of care (medical, surgical, intensive and services), type of contract (permanent or fixed-term) and years of working (<5, 6–9, 10–14, 15–19, 20–29 and >30). The level of statistical significance was set at 0.05. The results of the survey were analyzed with Stata 11 (StataCorp, College Station, TX, USA).

## 3. Results

### 3.1. Socio-Demographic and Professional Characteristics of the Sample

A total of 1849 professionals were surveyed, and 674 (36.45%) questionnaires were collected, but only 656 (35.48%) were considered valid. The responses of those who met the exclusion criteria or who had not completely filled in the MAWS and/or MBI sections were excluded. The questionnaire was validated by calculating the “Cronbach’s α” coefficient, and it showed a good level of internal consistency for the section of work motivation (α = 0.81) and burnout (α = 0.86).

The socio-demographic and professional characteristics of the study sample are summarized in Table 1. Most respondents were female (76.8%); 40.74% of participants were aged between 30 and 39. A total of 70.85% of participants had a university education (bachelor’s degree); around 10% of the sample specialized further (mostly master’s degree). Most respondents were nurses (87.04%); much smaller percentages of the sample represented, in decreasing order, laboratory technicians, physiotherapists, midwives and perfusion and radiology technicians. As for the type of healthcare activity offered, 38.21% of respondents belonged to the surgical area, 27.02% belonged to the medical area, 25.82% belonged to the intensive area and only 8.95% belonged to services. A large majority of participants (85.82%) had a permanent employment contract. At the time of the survey, the sample was distributed across the length-of-employment intervals, with 20.27% of respondents having worked for less than 5 years and 10.67% having worked for longer than 30 years.

### 3.2. Motivation at Work and Occupational Health

The participants revealed a high level of autonomous motivation (mean value 5.12, SD ± 1.04) and a low degree of controlled motivation (mean 2.50, SD ± 1.13) and demotivation (mean 1.61, SD ± 0.84). These aspects were also maintained when examining the four sub-categories of regulation types: a high level for intrinsic (mean 5.16, SD ± 1.03) and integrated motivations (mean 5.05, SD ± 1.24) and a low level for introjected (mean 3.04, SD ± 1.67) and external (mean 2.13, SD ± 1.10) ones (Table 2).

Data on self-rated levels of occupational well-being indicators are shown in Table 3. 

About half of the respondents (N = 342) rated their degree of general well-being as substantially unchanged compared to the previous year, while about 30% stated it has worsened. As for the depressive risk subscale, about 30% of respondents (N = 201) never felt depressed, while more than 10% felt depressed on half of the days and 5% declared feeling always depressed. Similar results were found for the emotional exhaustion subscale, where over 50% of respondents (N = 340) never felt exhausted. Furthermore, the majority of the sample (about 70%) were satisfied at work, while about 5% were completely dissatisfied. Only 18.32% (N = 120) wanted to leave the current care unit within two years, while 4.12% (N = 27) would have left the job. 

Considering the macro-categories of motivation at work, here, we found a clear association between scores on the autonomous motivation scale and self-rated indicators of occupational health (Table 4). HCWs rating low on the autonomous motivation scale reported lower levels of general health (*p* < 0.001), a high risk of depression (*p* < 0.001) and emotional exhaustion (*p* < 0.001), while those respondents who rated high on autonomous motivation were more work-satisfied (*p* < 0.001) and less willing to quit (*p* < 0.001). Controlled motivation was not significantly linked to any of the health’s indicators, while scores on the demotivation scale were positively associated with the intention to leave the care setting within 2 years.

Considering the sub-categories of regulation types (Table 4), scoring high on intrinsic regulation correlated with better general health (*p* = 0.012) and was positively associated with work satisfaction (*p* = 0.005). When integrated regulation was high, it reduced the risk of depression and emotional exhaustion (*p* < 0.001); in addition, scoring high on the integrated regulation scale was positively associated with job satisfaction (*p* < 0.001) and negatively associated with the intention to leave the care setting or the healthcare profession. Introjected regulation nourished the depressive risk, while no impact was found for the external regulation type (Table 4).

Interesting findings emerged when considering the impact of the various socio-demographic and professional factors on such associations (Table 5). 

In synthesis, the characteristics that worsened the occupational health indicators were operating in the intensive and surgical areas and having worked for 10–14 years (negatively affecting self-rated general health); being female and working as a nurse leader manager (nourishing depressive risk and reducing work satisfaction ratings) or midwife (increasing the emotional exhaustion load); and being older than 60 (higher exhaustion and a stronger drive to leave the setting and the job). By contrast, the characteristics associated with better occupational health were belonging to the age category of 30–39 years (favoring general health ratings), having obtained a master’s degree or the first-level university master (protecting from emotional exhaustion and depressive risk) and working as a perfusion technician (associated with lower scores on emotional exhaustion).

### 3.3. Motivation at Work and Burnout

Interesting findings emerged from the analysis of the association between work motivation/regulation scales and burnout indicators (Table 6). Briefly, controlled motivation, demotivation and external regulation were positively associated with EE; in contrast, autonomous motivation and related types of regulation (intrinsic and integrated) were negatively correlated with EE (*p* < 0.001). Demotivation was positively correlated with DE (*p* < 0.001), while autonomous (*p* < 0.001) and integrated efforts were negatively associated with this indicator (Table 6). 

In addition, autonomous motivation (*p* < 0.001) and integrated regulation (*p* < 0.001) were negatively linked to reduced professional achievement (RPA, *p* < 0.001), thus favoring the attainment of greater accomplishments at work.

Table 7 shows the possible impact of the socio-demographic and professional variables on the associations between work motivation and burnout. Variables nourishing the risk of burnout in HCWs were operating at surgical and intensive areas (moderating the risk for EE), having worked for a period of 10–14 years (specific for DE risk) and being a laboratory technician (increasing RPA risk). By contrast, other variables seemed to protect HCWs from the risk of burnout (Table 6): being female and being younger than 50 years (reducing risk of DE) and having obtained a second-level university master (protecting from RPA).

## 4. Discussion

The results of the present study confirm and extend data supporting the notion that the autonomous form of motivation at work must be considered as an important contributor to occupational well-being among healthcare personnel and as a relevant protective factor from burnout syndrome. Furthermore, our findings reveal that socio-demographic and professional variables can moderate this relationship. In our sample, high levels of autonomous motivation—i.e., finding work worthwhile, challenging, enjoyable and consistent with personal values—were associated with better general health ratings and with a lower risk of depression and emotional exhaustion and were correlated with higher work satisfaction and a weaker intention to quit the care unit or the healthcare job. The present data are in good agreement with what was observed in healthcare staff by other authors [4,11]. Moreover, we found that controlled motivation was not related to occupational health indicators, except for the interjected regulation—i.e., being driven primarily by the worry of feeling ashamed for not having achieved certain standards—that could predict a depressive risk [4]. Thus, as also pointed out in previous reports [4,11,17], HCWs who chose their job because they were authentically and autonomously motivated are healthier and more productive than those whose motivation is primarily controlled. In contrast, when demotivated or pushed by introjected regulation, HCWs are more vulnerable to developing apathy, indifference, a sense of irrelevance and the absence of involvement and a desire to change, all characteristics that worsen their occupational health status [3]. 

Here, we found that the associations between work motivation and occupational health are moderated by socio-demographic and professional variables. First, the care unit where HCWs operate turned out to be most relevant: working in the intensive and surgical areas had a negative impact on general health ratings in both Model 1 and Model 2 (thus moderating the associations of both motivation and related regulation types with general health). Similar results were reported by Liu et al. [32], who indicated surgical and intensive care units as work-related sources of dissatisfaction and malaise in healthcare personnel. In addition, the professional role was an important factor: in fact, being a midwife or working as a nurse leader manager negatively affected the association with occupational health indicators. The present finding that nurse leader managers are more prone to depressive risk, work-related dissatisfaction and turnover intention is in line with recent evidence showing an increasing trend in the occurrence of such cases [33], with negative repercussions on the degree of the involvement and the sense of belonging of the collaborators. Second, age was important: belonging to the age category of 30–39 years was found to favor general health ratings, whereas being older than 60 was linked with higher exhaustion and a greater intent to leave the care unit and the job [4]; these observations suggest that, at least in our study sample, younger workers are less motivated to quit and plausibly more protected from the risk of exhaustion. However, this seems at odds with the finding that having worked for 10–14 years negatively affected general health ratings in both Model 1 and Model 2, since such a period of service is compatible with the same age range showing a health-protecting role (30–39 years). Third, gender plays a role: being female nourishes depressive risk and reduces work satisfaction ratings, which is in line with several reports showing that the female gender among physicians displays a stronger link with depression than the male gender [4,34]. Fourth, the educational levels need to be considered: having obtained the first-level university master and the master’s degree protects HCWs from emotional exhaustion and depressive risk, in agreement with previous data showing that higher educational attainments allow for mental well-being and protect from burnout in healthcare professionals [35].

Work-related stress can be regarded as a key mediator linking job motivation, occupational health and the quality of the services provided. Evidence shows that in HCWs, unfavorable psychosocial job characteristics (high demands, low control and poor social support) exacerbate psychological distress (somatic complaints and emotional exhaustion), negatively affect job-related well-being (personal accomplishment, job satisfaction and work engagement) and increase the risk of burnout [36]. Importantly, the persistence over time of perceived stress is correlated with detrimental effects on workers’ health trajectories, with serious psychological and somatic consequences [37,38]. It is also well acknowledged that perceived stress in the workplace affects motivation, with levels of distress being inversely correlated with self-determined motivation [39]; specifically among HCWs, a significant inverse relationship between perceived stress and work motivation has been reported [40]. Here, the same study sample was used to investigate possible associations between work motivation and the risk of burnout by analyzing three different subscales: in general, the “bad” motivation/regulation types (i.e., controlled motivation, demotivation and external regulation) positively fed the emotional exhaustion (EE) and depersonalization (DE) subscales, while the “good” ones (i.e., autonomous motivations, including both intrinsic and integrated regulation) were found to be protective. This could mean that when motivated by self-determinate forces, HCWs are much less vulnerable to developing burnout syndrome, thus confirming previous reports bringing similar evidence [6]. In addition, here, we found that autonomous and integrated motivations were negatively linked to reduced professional achievement (RPA), meaning that HCWs who attained higher professional accomplishments are plausibly the ones being more autonomously motivated, as also reported in prior research [11]. Collectively, these observations suggest that, in the workplace, the transactional model of stress and the SDT motivational approach are complementary to each other and closely intertwined from both theoretical and practical perspectives. A high level of autonomous motivation is to be considered as an internal resource on which an individual can count, favoring the containment of distress and thus reducing prolonged stress-related negative consequences regarding individual general and occupational health.

Some of the sample’s socio-demographic and professional characteristics have been shown to have an impact on the observed associations between work motivation and burnout indicators. Being female, working as a nurse leader manager, operating in surgical and intensive care units, being older than 60 and having served for 6–14 years are all variables that nourished the link between low autonomous motivation and the risk of burnout syndrome. However, the fact that HCWs aged over 60 are the most vulnerable to develop burnout (in particular, the DE subscale) is disproved by previous studies [6] showing that nurses below 50 years of age are more likely to report high levels of burnout compared to older ones. Such apparent discrepancy, besides reflecting possible differences in the study samples recruited, can be interpreted in light of the STD [15,16]: human behavior can be self-determined, and every difference in work motivation stems from the dynamic and evolutive relationships between individuals, their working environment and their time of life. As such, behavior is the result of how everyone has progressively internalized his/her work, making it worthwhile and stimulating or not [11]. 

Surprisingly, the work seniority of 6–9 and 10–14 years was found to be associated with a higher risk of DE, while longer periods of service seemed protective. A young age is unlikely to contribute to this association, since we found that a young age (30–39 years) is actually protective and favors one’s occupational health status. Plausibly, then, the initial adjustment efforts that adapting to new job demands and a new job environment requires, with the massive physical, mental and emotional load involved, are so relevant that some workers cannot cope fully and immediately, thus risking developing and accumulating signs of burnout syndrome [41]. In this regard, it is desirable to plan and empower preventative programs aimed at potentiating the early tutoring for new recruits, so as to protect them from this risk. 

In our sample, the role played by gender appeared inconsistent. The female gender was more inclined to depressive risk in the regression model correlating work motivation with occupational health indicators (see Table 5), but being female turned out to be protective from the risk of depersonalization in the model correlating motivation with burnout (see Table 7). This ambiguity could be explained, at least in part, by considering the high complexity of the multifaceted concept of occupational health, which encompasses many different dimensions (including the “emotional exhaustion”, which is also an indicator of the burnout syndrome). In the first regression model, occupational health was considered in its whole, multidimensional complexity (which included a subscale of burnout), whereas the second regression model considered only burnout’s indicators. This observation emphasizes the possibility that using instruments and methodologies that are apparently very similar to each other may still produce markedly divergent results, and it calls for the need for paying great attention when planning research surveys in carefully selecting the appropriate indicators and psychometric instruments for investigating the targeted psychological dimensions.

The present survey suffers from some limitations: the design of the study is monocentric and transversal, thus hampering the generalizability of our results to different healthcare settings and preventing analyses of a causal relationship in the observed links. To evaluate the associations reported in this work more comprehensively and in depth, further research is warranted, including cross-sectional and prospective observational studies, but also intervention studies, which are to be conducted in a variety of healthcare settings and institutions.

Finally, and perhaps most importantly, the present study was carried out before the outbreak of the COVID-19 pandemic. Work attitudes such as work engagement, job satisfaction and work motivation have been shown to decline during the pandemic crisis in many job settings, including healthcare [42]. It is well known that the effects of inadequate staffing, a greater workload, extra shifts and physical exhaustion were exacerbated as hospitals surged with COVID-19 patients [25]. It is also well established that during the pandemic, nurses reported a high level of anger, depression and anxiety and a high desire to change their setting of care and, in most cases, type of work [43]. Moreover, nurses suffered higher levels of burnout, as they provided care during the pandemic [43]. Revolving the issue upside-down, it can be said that investigating the associations between work motivation and one’s occupational health status before the pandemic’s onset may have the merit of describing a more real, normal context of healthcare activity and workloads. Indeed, the present data may represent a valid reference standard for confronting the results of similar inquiries in healthcare professionals in post-pandemic periods, thus allowing for an accurate quantification of the burden of COVID-19 on occupational health in HCWs.

In our view, the results of the present study can help healthcare institutions to orient organizational efforts aimed at increasing the occupational health status of workers by promoting and supporting their autonomous motivation. Proactive planning and psychological interventions aimed at increasing support and promoting opportunities for professional growth, responsibility and autonomy are essential to attenuating work-related stress and to increasing work involvement and HCWs’ well-being [25]. In addition, increasing job satisfaction and reducing turnover intention among the personnel are advantages for the healthcare system in achieving organizational outcomes and saving costs [11]. 

## 5. Conclusions

Briefly, here, we showed that the autonomous form of work motivation positively correlates with occupational well-being and protects from burnout syndrome among healthcare personnel. Socio-demographic, educational and professional variables moderate this link: a younger age and higher educational levels fuel the association, whereas operating in intensive and surgical care units has a negative impact. Our observations underline the importance for healthcare organizations of promoting actions that reinforce autonomous motivation at work by providing support and opportunities for growth, improvement, responsibility and autonomy for workers, so as to increase work involvement and satisfaction and intent to stay and to attenuate work-related stress. Particular attention has to be paid to the needs of specific categories of HCWs, i.e., those working in particularly stressful care settings (surgical and intensive ones, or those covering the role of nurse leader manager) and older workers. 

In conclusion, the present data underline the need for the healthcare organizations to design and implement effective programs aimed at monitoring and potentiating work motivation as a means of preserving HCWs’ well-being and job satisfaction, thereby preventing negative consequences regarding the health trajectories of personnel and the quality of care delivered to patients.

## Figures and Tables

**Table 1:** Distribution of socio-demographic and professional characteristics of 656 HCW respondents.

Item		N	%	Item		N	%
Role (N = 656)	Nurse	571	87.04	Age (N = 653)	20–29	103	15.77
	Physiotherapist	23	3.51		30–39	266	40.74
	Laboratory technician	29	4.42		40–49	162	24.81
	Perfusionist	9	1.37		50–59	118	18.07
	Midwife	17	2.59		≥60	4	0.61
	Radiology technician	7	1.07	Care setting (N = 581)	Medical area	157	27.02
					Surgical area	222	38.21
Education (N = 652)	Bachelor’s degree	462	70.83		Intensive area	150	25.82
					Services	52	8.95
	First-level university master	114	17.88	Type of contract (N = 656)	Fixed-term	93	14.18
	Master’s degree	65	9.97		Permanent	563	85.82
	Second-level university degree	6	0.91	Years of working (N = 656)	<5 years	133	20.27
	Doctorate	5	0.76		6–9 years	110	16.77
Gender (N = 650)	Male	147	22.62		10–14 years	146	22.26
					15–19 years	81	12.35
	Female	503	77.38		20–29 years	116	17.68
					>30 years	70	10.67

**Table 2:** Scores in the Scale of Motivation At Work (MAWS) among HCW respondents.

Work Motivation	Mean	SD (±)	Subscale Item
Autonomous motivation	5.12	1.04	
Intrinsic regulation	5.16	1.03	1—Because I enjoy this work very much 4—Because what I am doing in my work is stimulating 5—Because the work I do is interesting
Integrated regulation	5.05	1.24	2—Because I am made for this job 3—Because I am fully fulfilled in this work
Controlled motivation	2.50	1.13	
Introjected regulation	3.04	1.67	9—Because otherwise I will feel bad about myself 10—Because otherwise I will feel ashamed of myself 11—Because I would feel guilty about not doing it 12—Because I have to prove to myself that I can
External regulation	2.13	1.10	13—To get others’ approval (e.g., supervisor, colleagues, family, clients…) 14—Because others will respect me more 15—To avoid being criticized by others 16—Because I risk losing benefits if I do not put enough effort into it 17—Because I risk losing my job if I do not put enough effort into it 18—Essentially for drawing a paycheck
Demotivation	1.61	0.84	6—I do not because I really feel I am wasting my time at work 7—I do not know why I am doing this job. It is pointless work 8—Honestly, I do little for my job

Note: Study participants were invited to respond to motivation items with: “Why do you involve in your work of providing patient care?”. Responses were given on a seven-point Likert scale with options ranging from 7 (“very true of me”) to 0 (“very untrue of me”). In our sample study, mean scores reveal relatively high levels of autonomous motivation and its sub-categories (intrinsic and integrated regulation) and low degrees of demotivation and controlled motivation and its sub-categories (introjected and external regulation).

**Table 3:** Distribution of the occupational well-being indicators in the study sample.

Item	Answer	N	%
General health (N = 656)	Better than one year ago	47	7.17
	A little bit better than one year ago	50	7.62
	Approximately the same as one year ago	342	52.13
	A little bit worse than one year ago	176	26.83
	Worse than one year ago	41	6.25
Depressive risk (N = 656)	Never felt	201	30.64
	Sometimes felt	357	54.42
	Felt on half of the days	68	10.37
	Always felt	30	4.57
Emotional exhaustion (N = 653)	Never felt	340	52.07
	Sometimes felt	252	38.59
	Felt on half of the days	40	6.13
	Always felt	21	3.21
Work satisfaction (N = 656)	Completely unsatisfied	29	4.42
	Unsatisfied	42	6.40
	Neither unsatisfied nor satisfied	123	18.75
	Satisfied	324	49.39
	Completely satisfied	138	21.04
Intention to leave the current practice in 2 years (N = 655)	Yes	120	18.32
	No	535	81.68
Intention to completely leave the healthcare profession in 2 years (N = 655)	Yes	27	4.12
	No	628	95.88

Note: The multiple-choice questions of the fourth section of the survey explored the occupational health status in the study sample. In particular, self-rated scores were given on the general health (from 1, “*better than 1 year ago*”, to 5, “*worse than 1 year ago*”) [28] and on several single indicators of the occupational health: risk of depression and emotional exhaustion (response options ranging from 1, “*never felt*”, to 5, “*always felt*”; [29]); work satisfaction (response options ranging from 1, “*completely unsatisfied*”, to 5, “*completely satisfied*”; [30]); intent to quit the care unit or the healthcare job in two years (binary choice: 1, “*yes, I want to quit*”, or 2, “*no, I am not willing to quit*”) [31].

**Table 4:** Effect of work motivation/regulation on six indicators of occupational health.

**(1) General health**					**(2) Depressive risk**				
**Variables**	**B**	**SE B**	**β**	***p***	**Variables**	**B**	**SE B**	**β**	***p***
**Model 1**					**Model 1**				
**Autonomous motivation**	**−0.188**	**0.040**	**−0.202**	**<0.001**	**Autonomous motivation**	**−0.206**	**0.032**	**−0.274**	**<0.001**
Controlled motivation	0.010	0.034	0.013	0.755	Controlled motivation	0.453	0.270	0.067	0.099
Demotivation	0.033	0.052	0.028	0.527	Demotivation	0.007	0.410	0.008	0.851
R^2^	0.125				R^2^	0.157			
F	2.760	Prob > F		<0.001	F	3.590	Prob > F		<0.001
**Model 2**					**Model 2**				
**Intrinsic regulation**	**−0.143**	**0.056**	**−0.151**	**0.012**	Intrinsic regulation	−0.038	0.044	−0.050	0.393
Integrated regulation	−0.059	0.046	−0.077	0.204	**Integrated regulation**	**−0.161**	**0.037**	**−0.259**	**<0.001**
Introjected regulation	0.014	0.027	0.026	0.590	**Introjected regulation**	**0.045**	**0.021**	**0.099**	**0.037**
External regulation	−0.006	0.042	−0.007	0.886	External regulation	−0.013	0.033	−0.019	0.683
R^2^	0.125				R^2^	0.167			
F	2.670	Prob > F		<0.001	F	3.710	Prob > F		<0.001
**(3) Emotional exhaustion**					**(4) Work satisfaction**				
**Variables**	**B**	**SE B**	**β**	***p***	**Variables**	**B**	**SE B**	**β**	***p***
**Model 1**					**Model 1**				
**Autonomous motivation**	**−0.228**	**0.029**	**−0.324**	**<0.001**	**Autonomous motivation**	**0.438**	**0.038**	**0.455**	**<0.001**
Controlled motivation	0.036	0.024	0.057	0.147	Controlled motivation	0.000	0.031	0.001	0.981
Demotivation	−0.004	0.038	0.902	−0.005	Demotivation	−0.088	0.048	−0.071	0.070
R^2^	0.213				R^2^	0.310			
F	5.200	Prob > F		<0.001	F	8.670	Prob > F		<0.001
**Model 2**					**Model 2**				
Intrinsic regulation	−0.025	0.040	−0.035	0.525	**Intrinsic regulation**	**0.147**	**0.052**	**0.151**	**0.005**
**Integrated regulation**	**−0.186**	**0.033**	**−0.319**	**<0.001**	**Integrated regulation**	**0.290**	**0.043**	**0.364**	**<0.001**
Introjected regulation	0.020	0.019	0.048	0.293	Introjected regulation	0.007	0.025	0.013	0.763
External regulation	0.012	0.029	0.019	0.665	External regulation	−0.021	0.039	−0.024	0.586
R^2^	0.227				R^2^	0.316			
F	5.430	Prob > F		<0.001	F	8.570	Prob > F		<0.001
**(5) Intent to leave the care setting within 2 years**					**(6) Intent to entirely leave the healthcare profession within 2 years**				
**Variables**	**B**	**SE B**	**β**	***p***	**Variables**	**B**	**SE B**	**β**	***p***
**Model 1**					**Model 1**				
**Autonomous motivation**	**0.048**	**0.017**	**0.126**	**0.005**	**Autonomous motivation**	**0.017**	**0.008**	**0.095**	**0.028**
Controlled motivation	−0.015	0.014	−0.045	0.287	Controlled motivation	0.008	0.007	0.049	0.232
**Demotivation**	**−0.051**	**0.022**	**−0.103**	**0.022**	Demotivation	−0.017	0.010	−0.076	0.080
R^2^	107				R2	0.164			
F	2.300	Prob > F		<0.001	F	3.790	Prob > F		<0.001
**Model 2**					**Model 2**				
Intrinsic regulation	0.003	0.023	0.008	0.892	Intrinsic regulation	−0.007	0.011	−0.039	0.505
**Integrated regulation**	**0.050**	**0.019**	**0.159**	**0.010**	**Integrated regulation**	**0.024**	**0.009**	**0.160**	**0.007**
Introjected regulation	0.001	0.011	0.006	0.908	Introjected regulation	0.010	0.005	0.090	0.059
External regulation	−0.026	0.018	−0.074	0.137	External regulation	−0.008	0.008	−0.045	0.339
R^2^	0.104				R^2^	0.170			
F	2.150	Prob > F		0.001	F	3.810	Prob > F		<0.001
**(1) General health**					**(2) Depressive risk**				
**Variables**	**B**	**SE B**	**β**	***p***	**Variables**	**B**	**SE B**	**β**	***p***
**Model 1**					**Model 1**				
**Autonomous motivation**	**−0.188**	**0.040**	**−0.202**	**<0.001**	**Autonomous motivation**	**−0.206**	**0.032**	**−0.274**	**<0.001**
Controlled motivation	0.010	0.034	0.013	0.755	Controlled motivation	0.453	0.270	0.067	0.099
Demotivation	0.033	0.052	0.028	0.527	Demotivation	0.007	0.410	0.008	0.851
R^2^	0.125				R^2^	0.157			
F	2.760	Prob > F		<0.001	F	3.590	Prob > F		<0.001
**Model 2**					**Model 2**				
**Intrinsic regulation**	**−0.143**	**0.056**	**−0.151**	**0.012**	Intrinsic regulation	−0.038	0.044	−0.050	0.393
Integrated regulation	−0.059	0.046	−0.077	0.204	**Integrated regulation**	**−0.161**	**0.037**	**−0.259**	**<0.001**
Introjected regulation	0.014	0.027	0.026	0.590	**Introjected regulation**	**0.045**	**0.021**	**0.099**	**0.037**
External regulation	−0.006	0.042	−0.007	0.886	External regulation	−0.013	0.033	−0.019	0.683
R^2^	0.125				R^2^	0.167			
F	2.670	Prob > F		<0.001	F	3.710	Prob > F		<0.001
**(3) Emotional exhaustion**					**(4) Work satisfaction**				
**Variables**	**B**	**SE B**	**β**	***p***	**Variables**	**B**	**SE B**	**β**	***p***
**Model 1**					**Model 1**				
**Autonomous motivation**	**−0.228**	**0.029**	**−0.324**	**<0.001**	**Autonomous motivation**	**0.438**	**0.038**	**0.455**	**<0.001**
Controlled motivation	0.036	0.024	0.057	0.147	Controlled motivation	0.000	0.031	0.001	0.981
Demotivation	−0.004	0.038	0.902	−0.005	Demotivation	−0.088	0.048	−0.071	0.070
R^2^	0.213				R^2^	0.310			
F	5.200	Prob > F		<0.001	F	8.670	Prob > F		<0.001
**Model 2**					**Model 2**				
Intrinsic regulation	−0.025	0.040	−0.035	0.525	**Intrinsic regulation**	**0.147**	**0.052**	**0.151**	**0.005**
**Integrated regulation**	**−0.186**	**0.033**	**−0.319**	**<0.001**	**Integrated regulation**	**0.290**	**0.043**	**0.364**	**<0.001**
Introjected regulation	0.020	0.019	0.048	0.293	Introjected regulation	0.007	0.025	0.013	0.763
External regulation	0.012	0.029	0.019	0.665	External regulation	−0.021	0.039	−0.024	0.586
R^2^	0.227				R^2^	0.316			
F	5.430	Prob > F		<0.001	F	8.570	Prob > F		<0.001
**(5) Intent to leave the care setting within 2 years**					**(6) Intent to entirely leave the healthcare profession within 2 years**				
**Variables**	**B**	**SE B**	**β**	***p***	**Variables**	**B**	**SE B**	**β**	***p***
**Model 1**					**Model 1**				
**Autonomous motivation**	**0.048**	**0.017**	**0.126**	**0.005**	**Autonomous motivation**	**0.017**	**0.008**	**0.095**	**0.028**
Controlled motivation	−0.015	0.014	−0.045	0.287	Controlled motivation	0.008	0.007	0.049	0.232
**Demotivation**	**−0.051**	**0.022**	**−0.103**	**0.022**	Demotivation	−0.017	0.010	−0.076	0.080
R^2^	0.107				R^2^	0.164			
F	2.300	Prob > F		<0.001	F	3.790	Prob > F		<0.001
**Model 2**					**Model 2**				
Intrinsic regulation	0.003	0.023	0.008	0.892	Intrinsic regulation	−0.007	0.011	−0.039	0.505
**Integrated regulation**	**0.050**	**0.019**	**0.159**	**0.010**	**Integrated regulation**	**0.024**	**0.009**	**0.160**	**0.007**
Introjected regulation	0.001	0.011	0.006	0.908	Introjected regulation	0.010	0.005	0.090	0.059
External regulation	−0.026	0.018	−0.074	0.137	External regulation	−0.008	0.008	−0.045	0.339
R^2^	0.104				R^2^	0.170			
F	2.150	Prob > F		0.001	F	3.810	Prob > F		<0.001

Note: Negative or positive associations between work motivation and occupational health indicators in HCVs were investigated using linear regression analyses. Model 1 explored the impact between the motivation macro-categories (autonomous and controlled motivation, demotivation) and each of the six indicators of occupational health. Model 2 examined the impact between the sub-categories of regulation types (intrinsic and integrated for autonomous motivation; introjected and external for controlled motivation). Both Model 1 and Model 2 were adjusted for the socio-demographic and professional characteristics of the sample: role, education, gender, age, care setting, type of contract and years of working.

**Table 5:** The role of socio-demographic and professional characteristics in the impact between work motivation/regulation and occupational health indicators.

**(1) General health**					**(2) Depressive risk**				
**Variables**	**B**	**SE B**	**β**	***p***	**Variables**	**B**	**SE B**	**β**	***p***
**Model 1**					**Model 1**				
30–39 years old	−0.306	0.158	−0.162	0.054	Nurse leader managers	1.808	0.718	0.101	0.012
10–14 years of working	0.356	0.182	0.160	0.051	Master’s degree	−0.879	0.391	−0.098	0.025
Surgical area	0.383	0.103	0.200	<0.001	Female	0.146	0.074	0.081	0.051
Intensive area	0.278	0.113	0.130	0.014					
R2	0.125				R2	0.157			
F	2.760	Prob > F		<0.001	F	3.590	Prob > F		<0.001
**Model 2**					**Model 2**				
30–39 years old	−0.320	0.162	0.170	0.050	Department manager	1.688	0.716	0.094	0.019
10–14 years of working	0.362	0.184	0.163	0.050	Master’s degree	−0.793	0.391	−0.088	0.043
Surgical area	0.382	0.104	0.200	<0.001	Female	0.156	0.074	0.087	0.036
Intensive area	0.285	0.113	0.134	0.012					
R2	0.079				R2	0.167			
F	2.670	Prob > F		<0.001	F	3.710	Prob > F		<0.001
**(3) Emotional exhaustion**					**(4) Work satisfaction**				
**Variables**	**B**	**SE B**	**β**	***p***	**Variables**	**B**	**SE B**	**β**	***p***
**Model 1**					**Model 1**				
Midwives	0.460	0.177	0.105	0.010	Department manager	−2.264	0.833	−0.098	0.007
First-level university master	−0.204	0.097	−0.085	0.036					
≥60 years old	1.414	0.506	0.120	0.005					
R2	0.213				R2	0.310			
F	5.200	Prob > F		<0.001	F	8.670	Prob > F		<0.001
**Model 2**					**Model 2**				
Perfusionist	−0.431	0.221	−0.076	<0.001	Department manager	−2.115	0.832	−0.092	0.011
Midwives	0.450	0.176	0.103	0.011					
First level of university master	−0.216	0.096	−0.089	0.025					
≥60 years old	1.394	0.497	0.118	0.005					
R2	0.227				R2	0.316			
F	5.430	Prob > F		<0.001	F	8.570	Prob > F		<0.001
**(5) Intent to leave the care setting within 2 years**					**(6) Intent to entirely leave the healthcare profession within 2 years**				
**Variables**	**B**	**SE B**	**β**	***p***	**Variables**	**B**	**SE B**	**β**	***p***
**Model 1**					**Model 1**				
Department manager	−0.814	0.376	−0.089	0.031	≥60 years old	−0.862	0.133	−0.284	<0.001
≥60 years old	−0.725	0.293	−0.112	0.014					
R2	0.107				R2	0.164			
F	2.300	Prob > F		<0.001	F	3.790	Prob > F		0.000
**Model 2**					**Model 2**				
Department manager	−0.767	0.378	−0.084	0.043	≥60 years old	−0.900	0.132	−0.296	<0.001
≥60 years old	−0.825	0.292	−0.128	0.005					
R2	0.104				R2	0.170			
F	2.150	Prob > F		0.001	F	3.810	Prob > F		<0.001

Note: The table depicts the impact of the socio-demographic and professional variables on the associations between work motivation/regulation and occupational health. Only data reaching the threshold for statistical significance are reported. As in Table 4, Model 1 concerned the macro-categories of motivation (autonomous and controlled and demotivation), while Model 2 concerned the regulation types (intrinsic and integrated for autonomous motivation; introjected and external for controlled motivation). Both Model 1 and Model 2 were adjusted for the socio-demographic and professional characteristics of the sample: role, education, gender, age, care setting, type of contract and years of working.

**Table 6:** Impact of work motivation/regulation on three subscales of burnout syndrome.

**EMOTIONAL EXHAUSTION (EE)**				
**Variables**	**B**	**SE B**	**β**	***p***
**Model 1**				
Demotivation	0.065	0.042	0.066	0.126
**Controlled motivation**	**0.081**	**0.028**	**0.118**	**0.004**
**Autonomous motivation**	**−0.175**	**0.033**	**−0.227**	**<0.001**
R^2^	0.188			
F	4.45			<0.001
**Model 2**				
**Intrinsic regulation**	**−0.110**	**0.045**	**−0.141**	**0.015**
**Integrated regulation**	**−0.077**	**0.037**	**−0.121**	**0.039**
Introjected regulation	0.019	0.022	0.040	0.392
**External regulation**	**0.077**	**0.034**	**0.107**	**0.024**
R^2^	0.185			
F	4.23			<0.001
**DEPERSONALIZATION (DE)**				
**Variables**	**B**	**SE B**	**β**	***p***
**Model 1**				
**Demotivation**	**0.132**	**0.045**	**0.126**	**0.004**
Controlled motivation	0.023	0.029	0.032	0.434
**Autonomous motivation**	**−0.153**	**0.035**	**−0.187**	**<0.001**
R^2^	0.175			
F	4.10			<0.001
**Model 2**				
Intrinsic regulation	−0.654	0.048	−0.079	0.178
**Integrated regulation**	**−0.110**	**0.040**	**−0.162**	**0.006**
Introjected regulation	0.002	0.023	0.003	0.946
External regulation	0.039	0.036	0.052	0.276
R^2^	0.165			
F	3.67			<0.001
**REDUCED PROFESSIONAL ACHIEVEMENT (RPA)**				
**Variables**	**B**	**SE B**	**β**	***p***
**Model 1**				
Demotivation	0.029	0.041	0.030	0.486
Controlled motivation	−0.025	0.027	−0.038	0.346
**Autonomous motivation**	**−0.196**	**0.032**	**−0.260**	**<0.001**
R^2^	0.194			
F	4.64			<0.001
**Model 2**				
Intrinsic regulation	0.035	0.043	0.046	0.415
**Integrated regulation**	**−0.217**	**0.036**	**−0.348**	**<0.001**
Introjected regulation	0.008	0.021	0.018	0.689
External regulation	−0.040	0.032	−0.057	0.215
R^2^	0.217			
F	5.15			<0.001

Note: Negative or positive associations between work motivation and burnout’s subcategories (emotional exhaustion, EE; depersonalization, DE; reduced professional achievement, RPA) in HCWs were investigated using linear regression analyses. As in Table 4 and Table 5, Model 1 concerns the impact of the macro-categories of motivation (autonomous and controlled and demotivation), while Model 2 concerns the impact of the regulation types (intrinsic and integrated for autonomous motivation; introjected and external for controlled motivation). Self-rated levels of EE, DE and RPA ranged from 1 (“low”) to 3 (“high”).

**Table 7:** The role of socio-demographic and professional characteristics in the impact of work motivation/regulation on burnout’s sub-categories (EE, DE and RPA).

**EMOTIONAL EXHAUSTION (EE)**									
**Variables**	**B**	**SE B**	**β**	***p***	**Variables**	**B**	**SE B**	**β**	***p***
**Model 1**					**Model 2**				
Surgical area	0.273	0.083	0.173	0.001	Surgical area	0.279	0.083	0.176	0.001
Perfusionist	1.00	0.249	−0.163	<0.001	Perfusionist	−0.976	0.249	−0.158	<0.001
R^2^	0.188				R^2^	0.185			
F	4.45	Prob > F		<0.001	F	4.23	Prob > F		<0.001
**DEPERSONALIZATION (DE)**									
**Variables**	**B**	**SE B**	**β**	***p***	**Variables**	**B**	**SE B**	**β**	***p***
**Model 1**					**Model 2**				
Intensive area	0.241	0.096	0.129	0.012	Intensive area	0.242	0.097	0.130	0.013
30–39 years old	0.379	0.135	−0.229	0.005	20–29 years old	−0.352	0.139	−0.213	0.012
40–49 years old	0.386	0.164	−0.199	0.019	30–39 years old	−0.361	0.170	−0.186	0.034
6–9 years of working	0.316	−0.152	0.146	0.039	6–9 years of working	0.312	0.155	0.144	0.045
10–14 years of working	0.380	0.155	0.195	0.014	10–14 years of working	0.373	0.158	0.191	0.018
					Female	−0.159	0.081	−0.082	0.048
R^2^	0.175				R^2^	0.165			
F	4.10	Prob > F		<0.001	F	3.67	Prob > F		<0.001
**REDUCED PROFESSIONAL ACHIEVEMENT (rPA)**									
**Variables**	**B**	**SE B**	**β**	***p***	**Variables**	**B**	**SE B**	**β**	***p***
**Model 1**					**Model 2**				
Second-level university master	0.945	0.383	−0.105	0.014	Second-level university master	−0.843	0.380	−0.094	0.027
					Laboratory technician	0.957	0.198	0.265	<0.001
R^2^	0.194				R^2^	0.217			
F	4.64	Prob > F		<0.001	F	5.15	Prob > F		<0.001

Note: The table depicts the impact of the socio-demographic and professional variables on the associations between work motivation/regulation and three subscales of burnout syndrome (emotional exhaustion, EE; depersonalization, DE; reduced professional achievement, RPA). Only data reaching the threshold for statistical significance are reported. As in previous tables, Model 1 concerns the macro-categories of motivation (autonomous and controlled and demotivation), while Model 2 concerns the regulation types (intrinsic and integrated for autonomous motivation; introjected and external for controlled motivation). Both Model 1 and Model 2 were adjusted for the socio-demographic and professional characteristics of the sample: role, education, gender, age, care setting, type of contract and years of working.

## Data Availability

The authors own this data and agree to make the materials and data available to the journal and other researchers upon request.
